# Proarrhythmia Induced by Propafenone: What is the Mechanism?

**Published:** 2010-06-05

**Authors:** Francisco Femenia, Jorge Palazzolo, Mauricio Arce, Martin Arrieta

**Affiliations:** 1 Unidad de Arritmias, Departamento de Cardiología, Hospital Espanol de Mendoza, Argentina; 2 Servicio de Cardiología, Hospital El Carmen, Mendoza, Argentina

**Keywords:** atrial flutter, propafenone, proarrhythmia, wide complex tachycardia

## Abstract

Propafenone, a Class IC antiarrhythmic drug, is an orally active sodium channel-blocking agent. It is effective in supraventricular tachyarrhythmias and is particularly useful in converting atrial fibrillation to sinus rhythm. In therapeutic doses, it may cause non-cardiac and cardiac toxicity, including  proarrhythmia.

## Case Report

**Figure 1:**
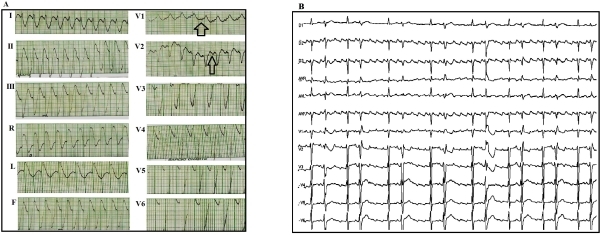
12-lead electrocardiogram A: Wide QRS complex tachycardia. Heart rate at 150 bpm, with right bundle branch block morphology, left axis deviation and visible atrial activity in V1 V2 (arrow). B: Typical atrial flutter with variable AV conduction.

**Figure 2:**
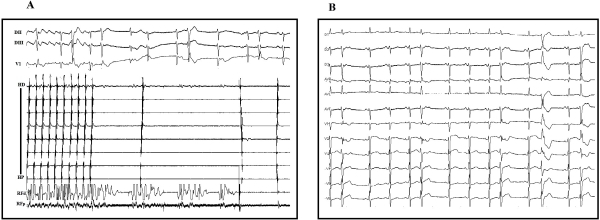
A: Radiofrecuency ablation. Termination of atrial flutter during RF application. Normal sinus rhythm is restored.  B: Normal sinus rhythm after RF ablation.

A 69 year-old female with hypertension and paroxysmal atrial fibrillation presented with palpitations and dyspnea. Her current medications included enalapril, atenolol and amiodarone. Physical examination showed heart rate 140 bpm and irregular. Blood pressure was 140/90 mm Hg. There was no evidence of heart failure. She was initially treated with verapamil 5 mg (IV) for rate control and propafenone 600 mg (orally) for pharmacological cardioversion. Being admitted, the patient reported rapid palpitations, dizziness and increased dyspnea. A 12-lead electrocardiogram (ECG) showed self-limited wide QRS complex tachycardia (Figure 1A) at a heart rate of 150 bpm with right bundle branch block (RBBB) morphology in lead V1 and left axis deviation. Ventricular tachycardia was suspected. To elucidate the mechanism of the wide QRS complex tachycardia, the patient was referred to our institution for an invasive EP study. However, before the study and after propafenone discontinuation, another 12-lead ECG showed typical right sided isthmus dependent atrial flutter (AFL) with variable AV conduction (Figure 1B). This rose the suspicion of a proarrhythmic tachycardia induced by propafenone. Typical flutter was confirmed and RF ablation was successfully performed. (Figure 2A-B).

## Discussion

Propafenone is a class IC antiarrhythmic drug that is commonly used for atrial fibrillation treatment [1]. However, as shown in this case, its use is not complication free. These agents act by slowing sodium influx into myocytes through voltage gated sodium channels, and are referred to us as sodium-channel blockers. They differ in their affinity and duration of binding to the sodium channel, in their effects on potassium and calcium channels, and in other effects such as anticholinergic properties. Class IC agents are the most potent sodium-channel blockers but do not affect potassium channels; therefore these agents cause QRS prolongation without QT prolongation[2].

Metabolism of propafenone is mediated by the cytochrome P-450 (CYP) isoenzyme system, including CYP2D6 (major metabolic pathway), CYP1A2 and CYP3A4. Patients should be monitored and dosage of propafenone should be reduced accordingly when the drug is used concurrently with other inhibitors of CYP2D6 (e.g.: quinidine) and CYP1A2 (e.g.: amiodarone) because plasma concentrations may increase. In therapeutic doses, propafenone may cause non-cardiac and cardiac toxicity including: agranulocytosis, reactive airway disease, hemolysis, drug fever, ataxia, hypotension, prolonged QRS duration, ventricular arrhythmias, depressed mental status, seizures and sudden death. The overall incidence of proarrhythmia with propafenone is unknown. It was reported as high as 5-10% [3]. Class 1C antiarrhytmic drugs significantly slow conduction velocity of the atrial tissue with moderate effects on the prolongation of refractory periods, including the atrioventricular node. Consequently, slowing the AFL cycle length may lead to 1:1 atrioventricular conduction, and widening of the QRS complex [4].

The ventricular response during AFL is determined by the refractory period of the AV node, the degree of concealed conduction within the node, and the status of the autonomic tone. The association of 1:1 AFL with high catecholamine levels has been previously described. Sympathetic tone might play an important role in the occurrence of 1:1 AFL [5]. Kawabata et al [5] showed that 1:1 AFL can occur during physical activity linking to a life-threatening haemodynamic status (syncope, presyncope and shock). In the differential diagnosis, one must exclude ventricular tachycardia and extreme aberrant conduction, mimicking ventricular tachycardia. This exercise is not always easy because of the QRS widening, high ventricular rate and hemodynamic intolerance. Vagal maneuvers can sometimes increase the degree of atrioventricular block unmasking atrial flutter and usually, all the conventional criteria for ventricular tachycardia are not present.

Propafenone can produce sustained monomorphic ventricular tachycardia, which is characterized by a marked increase in QRS duration and not associated with QT interval prolongation. Its heart rate is relatively slow but tends to be incessant. The mechanism of this proarrhythmic effect is likely to be due to a delay of conduction in the His-Purkinje system. This effect may encourage reentry by allowing extra time for refractory tissue to recover, thus allowing it to be reexcited  [6].

## Conclusion

Propafenone is a useful drug for the treatment o atrial fibrillation. It has proarrhythmic effects, such as 1:1 conducted atrial flutter and ventricular tachycardia. Rapid recognition of these complications allows discontinuing the medication and resolution of the proarrhythmia.
